# Understanding the relationship between eye disease and driving in very old Australian women: a longitudinal thematic evaluation

**DOI:** 10.1186/s12886-022-02506-8

**Published:** 2022-06-24

**Authors:** Jennifer White, Mitiku Teshome Hambisa, Dominic Cavenagh, Xenia Dolja-Gore, Julie Byles

**Affiliations:** Centre for Women’s Health Research, College of Health Medicine and Wellbeing, Faculty of Health and Medicine, University of Newcastle, Callaghan, Australia

**Keywords:** Driving, Ophthalmology, Qualitative research, Women, Vision

## Abstract

**Background:**

Over recent decades an increasing number of adults will retain their driver’s licenses well into their later years. The aim of this study was to understand and explore the experience of driving and driving cessation in very old Australian women with self-reported eye disease.

**Methods:**

An interpretative qualitative study. Participants were from the Australian Longitudinal Study on Women’s Health (cohort born in 1921–26), a sample broadly representative of similarly aged Australian women. Responses to open-ended questions were analysed using an inductive thematic approach, employing a process of constant comparison.

**Results:**

Qualitative data were from 216 older women with eye disease who made 2199 comments about driving, aged between 70 and 90 years depending on the timing of their comments. Themes included: (1) Access to treatment for eye disease promotes driving independence and quality of life; (2) Driving with restrictions for eye disease enables community engagement and (3) Driving cessation due to poor vision leads to significant lifestyle changes.

**Conclusions:**

Key findings highlighted driving cessation, or reduction, is often attributed to deterioration in vision. The consequence is dependence on others for transport, typically children and friends. Access to successful treatment for eye disease allowed older women to continue driving. We posit that occupational therapists can play an essential role in promote driving confidence and ability as women age.

Trial registration: Not applicable.

## Introduction

Consistent with international profiles, the proportion of older people in Australia is increasing and by 2057 it is estimated that people over 65 years will account for 22% of the Australian population (8.8 million people) [1]. The percentage of older adults who will retain their driver’s license to an older age is increasing and in 2015, 69% of Australians over 80 years were still driving, an increase of 59% since 2007. In Western countries driving is important for community access and participation [2] and is now considered an activity of daily living (ADL) [3]. The practical and emotional benefits of driving and the association with quality of life have been well documented [3, 4]. Overall, driving enables people to fulfil ADLs such as banking and shopping, and promotes social and community engagement [5]. Furthermore, driving is associated with feelings of independence [6], having a sense of control in life [7] and self-identity [5].

Growing evidence highlights the association between driving cessation and reduced health care utilisation [8], depression [9] and nursing home admission [10, 11]. Driving is a highly complex task that requires physical, cognitive, behavioural, visual and sensory-perceptual abilities [12]. All of these abilities are at risk of age-related decline and can negatively affect capacity to drive in different ways [13].

Within Australia, 9.4% of the population aged 55 years and over experiences a major eye disease causing visual impairment such as macular degeneration (MD), cataract, glaucoma and diabetic retinopathy [14]. The risk of these conditions increase with age and the prevalence is higher in older age groups. Impaired vision has implications for safe driving such as the ability to judge oncoming traffic, pedestrians and road signs, all of which present at varying height levels, with differing visual contrast and speed [15]. With treatment many people continue to drive with eye disease until the advanced stages of the disease [16, 17] thus promoting quality of life and independence [18, 19]. With age, and associated disabilities, drivers are likely to modify their driving by limiting the distance travelled [15] and avoiding challenging driving situations [15, 20–22] in order to feel safe prior to ceasing driving [23]. For example previous research has demonstrated that some people with cataracts self-regulate their driving while waiting for cataract surgery [24]. Similarly drivers with glaucoma are likely to restrict night driving [25] and drivers with MD are more likely to avoid driving over longer distances, at night and in unfamiliar conditions [26]. Additional research highlights a gender differences such that older women are more likely to report difficulties with driving [27], limit where they drive [28], drive shorter distances [9] and only drive in good weather [29]. A study by Foley et al [30], exploring life expectancy and driving life expectancy among 4699 people (aged 70–74 years) over a two year period and identified that women drivers were more likely to rely on others for transport in the last 10 years of life, in comparison to men who only relied on others for the last 7 years [30]. The authors posit that because of differences in life expectancy, women require more years of support for transportation, on average, than men after age 70 [30].

This current study is part of a broader research progm exploring the predictors of driving among women as they age from 76 to 81 to 85–90 years using the 1921–26 cohort of the Australian Longitudinal Study on Women’s Health (ALSWH) data from survey waves three to six, based on the World Health Organization's (WHO) Healthy Ageing framework [31]. In brief, we identified that medical conditions such as diabetes, stroke, poor vision, and need of assistance for daily tasks due to long term chronic illness, disability or frailty have negative associations with driving over time [31]. Using the 36-Item Short Form Health Survey (SF-36) [32] we also identified that non-drivers scored consistently lower on SF-36 subscale scores for physical functioning, social functioning, and general health sub score compared to drivers over time in these participants [31]. The aim of this current paper is to understand and explore the qualitative experience of driving and driving cessation in Australian women in the oldest cohort (1921–26) with self-reported eye disease who responded to free text survey comments.

## Study design

The ALSWH is a population-based observational study of > 57,000 women in four age cohorts. Details of the study including design, recruitment methods and national representativeness are described elsewhere [33, 34]. The ALSWH examines demographic, social, physical, psychological, and behavioural variables and their effect on major aspects of women's health and well-being and health service use. We analysed free-text comments among women who reported eye surgery, cataracts, macular degeneration and glaucoma in the oldest cohort (born 1921–26). Research incorporating the ALSWH surveys are approved by the University of Newcastle and the University of Queensland’s Human Research Ethics Committees (Approval Numbers H-076–0795 + H-2012–0256, and 2,004,000,224 + 2,012,000,950, respectively).

## Methods

An interpretive qualitative approach. Of the 12,432 women in the ALSWH oldest (born in 1921–26) we examined the longitudinal experience of emergent or ongoing eye disease and driving status based on free text comments made in main surveys (conducted three yearly from 1996–2011) and subsequent six-month follow-up surveys that were returned before July 2020. This included free text comments made by women who did not report eye disease or treatment and self-driving in the survey questions but made references to eye disease in their free-text comments. The study was informed by the Consolidated criteria for reporting qualitative research (COREQ) checklist [35]. In addition supporting quantitative data on physical and psychosocial functioning were examined to provide context to the qualitative findings. The ability to explore capture longitudinal comments allowed us to present patterns of driving overtime due to interventions for eye disease and the experience of other health conditions.

### Data

Quantitative demographic, chronic disease and health related quality of life variables are taken from ALSWH main surveys 1 to 6 (1996 to 2011) and the first six-month follow-up survey (late 2011). These surveys cover age ranges from 70–75 to 85–90. Main method of transport and the experience of chronic conditions were taken from the question ‘In the last three years have you been diagnosed with or treated for:’ with options ‘Cataracts’, ‘Macular degeneration’ and/or ‘glaucoma’. Women were also asked if they had any eye surgeries, including cataract surgery in the last three years. These conditions were treated as enduring meaning that once a women reported the condition on one survey, she was considered to have the condition for all subsequent surveys. The SF-36-item Short Form Survey was taken from Medical Outcomes Survey and used to measure health related quality of life [36]. General health, physical functioning and mental health subscales were calculated at each survey [36].

At the conclusion of each ALSWH survey, participants are asked “Have we missed anything?” and are invited to record free-text comments on their health and any other topics that they felt the survey did not adequately include.

### Analysis

Of the 12,432 women in the oldest cohort (born 1921–26), 8,550 women provided at least one comment at any survey point resulting in 35,900 comments. Of these 8,550 women 4,610 responded in the affirmative to survey questions about eye disease or having eye surgery and 4458 ever reported driving themselves. In total the number of women reporting eye disease or treatment and who ever reported driving was 2,783. Of these women, 163 provided at least one comment related to eye disease and driving. Additionally, comments made by 53 women who did not report eye disease or treatment and self-driving in the survey questions were included and found to be relevant since they referred to their experience of eye disease in their free-text comments. In the final data set, there were 2199 free text comments from 216 women relating to eye disease and driving. Each free text response was transcribed verbatim with identifying details removed. Microsoft Excel (Microsoft excel, 2016) was used to manage the survey data and compare the responses both cross-sectionally (according to each survey point) and longitudinally (following a single woman over time). Since participants’ comments varied in length and depth of information, we employed a qualitative content analysis, allowing for large amounts of data to be reduced to concepts that fit with the research questions [37]. Initially, all transcribed data were read by the principal investigator (JW) several times in order to obtain familiarity within-case analysis information. Codes were deductively derived into initial codes inspired by the focus of study and the experience of driving with eye disease or not. Detailed analytic memos of emergent issues and patterns were kept throughout the coding process. In the second analytic phase, a cross-case analysis of patterns emerging over time was conducted. Patterns that emerged from the cross-sectional and constant comparative analysis across points of time were thematically grouped. To avoid bias, data were analysed separately by two independent researchers from different professional backgrounds (JW an occupational therapist and JB a gerontologist). Given the large amount of data, we engaged a third researcher (MTH) to independently reviewed 10% of the transcripts to uphold validity of coding. Any disagreement was discussed to reach consensus of themes. We also mapped longitudinal comments and developed patterns of driving cessation due to interventions for eye disease and the experience of other health conditions.

Proportions of categorical quantitative variables and means and standard deviations for continuous variables (SF-36 scale scores) were tabulated for each survey for the women selected in qualitative analysis. Note that not all questions were asked at each survey, so these variables are not available at each survey. Also, due to attrition not all 216 women completed each survey. Statistics are cross sectional at each survey based on those who are eligible at that time point.

## Results

The characteristics of the 216 women providing relevant comments for qualitative analysis are shown in Table 1. Between 1996 and 2011, these women generally moved from being partnered to being single. While most of the women lived in a home, and still lived in a home in 2011, there was a general move into retirement villages/Nursing homes/hostels. By 2011, > 60% of women still drove themselves for their main form of transportation. There were high levels of eye related chronic diseases (cataracts, macular degeneration, glaucoma) over time. Almost 70% of women who returned a survey in 2011 had had eye-related surgery previously. While scale scores for mental health remained relatively high between 1996 and 2011, there was an overall reduction in general health and physical functioning.

**Table 1:** Demographic, chronic disease and health related quality of life measures across survey main waves 1–6 and the first 6 monthly follow-up survey

	**Survey 1**	**Survey 2**	**Survey 3**	**Survey 4**	**Survey 5**	**Survey 6**	**6MF1**
	*n* = *216*	*n* = *210*	*n* = *210*	*n* = *207*	*n* = *188*	*n* = *166*	*n* = *166*
**Categorical Measures (column %)**							
**Partnered status**							
*missing*	1.39	0.95	0.48	0.97	1.06	0.00	1.20
*Single*	44.91	48.10	59.52	68.12	75.00	81.33	81.93
*Partnered*	53.70	50.95	40.00	30.92	23.94	18.67	16.87
**Housing situation**							
*missing*	3.24	1.90	0.48	1.93	2.66	2.41	1.20
*House*	84.26	82.86	80.00	72.46	67.55	64.46	62.05
*Apartment*	10.65	10.48	12.86	14.01	13.83	12.65	13.86
*Retirement/Nursing/Hostel*	1.85	4.76	6.67	11.59	15.96	20.48	22.89
**Transportation**							
*missing*			5.24	6.76	9.04	9.04	3.61
*Other transport*			10.00	14.01	19.68	25.90	31.33
*Drive themselves*			84.76	79.23	71.28	65.06	65.06
**Cataracts (ever)**							
*missing*				0.00	0.00	0.00	0.00
*No*				64.25	48.94	34.34	34.34
*Yes*				35.75	51.06	65.66	65.66
**Any eye surgery (inc. cataract) (ever)**							
*missing*			4.29	0.48	0.00	0.00	0.00
*No*			71.90	54.11	36.17	24.70	25.30
*Yes*			23.81	45.41	63.83	75.30	74.70
**Macular degeneration (ever)**							
*missing*					1.06		1.20
*No*					77.13	69.88	69.28
*Yes*					21.81	30.12	29.52
**Glaucoma (ever)**							
*missing*					1.06	0.00	1.20
*No*					82.98	80.72	78.92
*Yes*					15.96	19.28	19.88
**Continuous Measures (Mean (Std. Dev.))**							
**General health score (SF36)**	73.11 (17.89)	72.29 (18.12)	71.37 (19.37)	68.15 (19.57)	66.36 (20.49)	64.74 (19.8)	61.07 (19.87)
**Physical functioning score (SF36)**	73.58 (21.34)	71.97 (21.69)	68.58 (23.94)	64.25 (26.49)	56.97 (25.49)	48.85 (26.83)	48.64 (25.31)
**Mental health score (SF36)**	81.53 (14.12)	81.61 (14.45)	81.83 (13.83)	81.14 (14.5)	80.76 (15.24)	81.11 (14.35)	78.73 (15.97)

. = Survey measure was not included in that survey wave

6MF1 = 1.^st^ 6 monthly follow-up survey

Of the 216 participants who provided one or more free text comments, we identified three broad patterns of driving over time (see Table 2). The first involves participants who continued driving, and who described access to treatment of eye disease and the instigation of driving restrictions as vision deteriorated. The second involves participants who ceased driving, and who attributed this to deterioration of vision. The third involves participants experiences of eye disease, but who attributed the cessation of driving to other health conditions (Table 3).

**Table 2:** Patterns of driving with eye disease based on self-reported comments

Condition	Driving continuation over time *N *= 63	Driving cessation due to poor vision *N* = 73	Driving with poor vision but ceases due to other health conditions *N* = 80
Macular Degeneration (MD), *n* = 83	18% (*n* = 15)	52% (*n* = 43)	30% (*n* = 25)
Cataract, *n* = 95	38% (*n* = 36)	11% (*n* = 10)	52% (*n* = 49)
Glaucoma, *n* = 31	39% (*n* = 12)	48% (*n *= 15)	13% (*n* = 4)
Other, *n* = 7		72% (*n* = 5)	29% (*n* = 2)

**Table 3:** Case studies of driving continuation or cessation

Trajectory	Case	Survey 1–3	Survey 4	Survey 5	Survey 6	Survey 6MF
Driving continuation over time	Case 1 (S25)	Driving	Driving	Driving	Driving Survey 6: Macular degeneration	Driving Survey 6MF2: I shall need to have two cataracts removed this year. I am still able to drive
Driving continuation over time	Case 2 (S131)	Driving	Driving	Driving	Driving Survey 6MF2: Cataract surgery	Driving Survey 6MF5: Glaucoma Survey 6MF8: Driving restrictions
Driving cessation due to poor vision	Case 1 (S23)	Driving Survey 1: Hip and knee replacements	Driving	Driving	Driving	Ceased driving Survey 6MF1: Macular degeneration Survey 6MF3: Macular degeneration has worsened and now unable to drive
Driving cessation due to poor vision	Case 2 (S110)	Driving	Driving Survey 4: Cataract surgery	Driving	Driving Survey 6: Macular degeneration	Ceased driving Survey 6MF1: Vision deteriorating Survey 6MF2: Vision impaired
Driving cessation due to other health conditions	Case 1 (S49)	Driving Survey 3: Radical Mastoidectomy	Driving	Driving	Driving	Ceased driving Survey 6MF1: Angina and bowel cancer Survey 6MF4: Angina Macular degeneration Survey 6MF7: Trouble breathing. I found that I was getting flustered in traffic
Driving cessation due to other health conditions	Case 2 (S199)	Driving	Driving Survey 4: Mitral valve prolapse	Driving	Driving	Ceased driving Survey 6MF1: Heart condition, two Cataracts treated, two Knee Replacements Survey 6MF5: Cancerous lesions on legs Survey 6MF9: Fall, fractured the neck of the femur, ceased driving

6MF1 = 1^st^ 6 monthly follow-up survey; Survey 6MF2: 2^nd^ 6monthly follow-up survey; 6MF3: 3rd 6 monthly follow-up survey; Survey 6MF4: 4^th^ 6monthly follow-up survey; Survey 6MF5: 5^th^ 6 monthly follow-up survey; Survey 6MF9: 9^th^ 6 monthly follow-up survey; 6MF12: 12^th^ 6 monthly follow-up survey

### Themes

Our analysis takes into consideration that the prevention and management of chronic eye conditions being an important part of overall health that promotes driving. Free text comments captured participants experience of deteriorating eyesight due to eye disease and its impact on driving capacity and subsequent quality of life. Our findings identified three themes:

1. Access to treatment for eye disease promotes driving independence and promotes quality of life
2. Driving with restrictions for eye disease still enables community engagement
3. Driving cessation due to poor vision leads to significant lifestyle changes

Illustrative quotations are identified by survey point and the nature of eye disease.

#### Access to treatment for eye disease promotes driving independence and promotes quality of life

Participants commonly reported the onset and/or ongoing experience of MD, cataract, glaucoma and other eye diseases. Central to this theme was access to specialist treatment for eye disease and the link to ongoing adequacy of eyesight that ensured independence with driving.

*I have macular degeneration and receive regular injections of Lucentis—so far am allowed to drive. (Survey Identification number (S)52, macular degeneration (MD))*

Another woman reiterated:

*My macular degeneration is being closely monitored. I have Lucentis Injections in both eyes monthly – but I am still reading writing and driving, (S47, MD)*

While participants reported that treatment wasn’t always as effective as they hoped they were pleased to be able to continue to drive for as long as possible.

*My biggest problem is my dry eyes…but I passed my driving test with a good report. (S31, MD)*

Another woman said:

*I had cataract operations on both eyes—before things got to the stage where I could not pass the eye test for my annual drivers’ licence. (S133, cataract (C))*

Some participants’ eye conditions were diagnosed late, or they experienced complications from their eye treatment which led to concerns about their continued ability to keep driving.

*About two years ago I had iritis which was not picked up. As a result, I have permanent damage to my eyes. This has curtailed my activities, but I can still I drive. (S10, C)*

*Unfortunately, my eye became badly infected. Never have I suffered such pain. Needless to say, no activities since it happened. I am hopeful that I shall be able to drive again. (S5, MD)*

Successful treatment for long term eye disease meant that many participants were able to continue driving, as well as other valued activities.

*I am 77 years old and have had trouble with my eyes particularly distance. Recently I had cataract operations on both eyes and now I drive my car without glasses too. (S27, C)*

*Cataract operations on both eyes were great success and I can read normal print without glasses. I still drive my car around local area. (S45, C)*

Many participants reportedly experienced improved vision after treatment, especially cataract surgery, which subsequent lead to feelings of greater confidence with driving.

*Removal of cataracts on both eyes made a huge improvement in general confidence—as well as enabling me to drive again at night, read music and small type with ease and more. (S62, C)*

*Since the last survey I have had cataract operations (both eyes) which has made me much more confident about driving, especially at night. (S66, C)*

All participant expressed that driving contributed to greater feelings of meaning in life. For many the ability to drive was linked with independence and feelings of *“freedom of lifestyle.” (S110, C).*

*What more could I want at my age. My driving license has been renewed for another 3 years, so I am independent. I am making the most of it while I can. (S50, MD)*

Indeed, many participants expressed concern about the prospect of losing their license in the future and how they would *“manage” (S164, G*) when driving was no longer possible.

*I was recently shocked and disappointed to find that my peripheral vision is deteriorating. Should it continue to do so -to the extent that I can no longer hold a driving licence, then my lifestyle and my independence will be severely affected. (S183, C)*

Many participants reported that driving allowed them to care for loved ones and provide a service to the community.

*I had cataracts removed from my eyes in July and I can now see without glasses. I care for my husband who is 97 years old, and I do all the driving and shopping. (S117, C)*

*Two cataracts forming, glaucoma being treated and macular degeneration. I do a lot of hospital and nursing home visitation and taking a few people out for short drives. (S210, C)*

Overall, many participants valued passing their eyesight and driving tests as they aged.

*Macular degeneration of eyes. Right eye: Ok but now developing a cataract. Left eye: Centre blindness due to scar on retina, but still managed to pass (but only just) eyesight test for driving license. (S127, MD)*

#### Driving with restrictions for eye disease enables community engagement

All participants valued the opportunity to keep driving despite the experience of formal or self-initiated driving restrictions. Indeed, many participants relied on the opinion of the others such as a family member or their local doctor when deciding when to consider ceasing driving or implementing restrictions.

*I realise I am slowing down. However, my daughter does not query my driving, nor does my doctor. (S22, C)*

Many participants reportedly self-initiated restrictions such as only driving in the daytime, in local areas or in less demanding situations as their eyesight deteriorated.

*Macular degeneration but ongoing injections still holding the sight in one eye. Still driving but only when I know exactly where I'm going as I can't read the signs early enough. Haven't driven at night for some years now. (S70, MD)*

*My health remains stable. I continue to be involved in many activities. Even though I have a full driver licence I choose not to drive at night. (S102, C)*

*I have had an eye operation to remove a Cataract. Waiting to get the second eye done, I gave up driving. I don't think I will drive again because the heavy haulage trucks on the road. (S204, MD)*

Other participants reported the driving restrictions were implemented following formal driving tests.

*Macular degeneration in my left eye. I passed my driving test – restricted to 100 km distance. (S120, MD)*

Alongside reported deterioration in eyesight participant also reported that advancing age and other health conditions caused them to self-restrict their driving.

*I have just turned 90! I still drive my car—but not at night. Nor do I go far—just to near suburbs. (S22, C)*

*I have recently developed several fractures in the spine causing severe pain in lower spine and buttocks. I have eased driving my car because of drugs for pain. (S4, C)*

As participants eyesight deteriorated, many reportedly relied on family and friends to drive in complex driving scenarios.

*Neither my husband nor I like to drive at night, so if necessary, a family member comes to the rescue. (S29, C)*

#### I drive myself when the distance is short reason is simple, otherwise my daughter drives me. (S205, MD)

For those participants who reported they temporarily ceased driving while waiting treatment there was the common experience of reduced activity and the need for assistance with ADLs.

*I am waiting for an operation to remove cataracts on my eyes. This means that I am unable to drive at present. This limits my mobility. (S105, C)*

*I have had an operation on my eye. A cancer on my lower eyelid and plastic surgery from my top lid. I have not been able to drive for over a month so have needed help with shopping. (S130, other)*

#### Driving cessation leads to significant lifestyle changes

Over time many participants reported they were no longer able drive due to poor vision or other health conditions such as pain, cognitive decline, poor mobility, and cancer.

*I have glaucoma and was operated on for it in 1963 and could not pass my eye test to continue driving when I turned 75 last year. It was a blow to me but am getting used to the situation now. (S182, G (glaucoma))*

*I have recently developed several fractures in the spine causing severe pain in lower spine and buttocks. Ceased driving my car because of drugs for pain. (S4, C)*

Participants reported varied reasons for self-initiated driving cessation such as increasing age, loss of confidence and *“feeling flustered in heavy traffic”* (S50, MD), or finding driving difficult.

*Due to macular degeneration in one eye- during the past three- six month, I have found driving more difficult- despite getting an unrestricted license in the past twelve months- as a result, I have had to give up driving. (S41, MD)*

*My eyesight seems greatly affected- I have given up my driving licence as I am now 90 years old and not quite so confident (S177, C)*

Many participants who ceased driving due to poor vision in an earlier survey later reported being *“legally blind”* (S140, AMD) in a later survey. The experience of being legally blind was commonly linked with not being able to continue valued activities such as reading and craft.

*I have had to sell my car, have had to give up sewing, knitting, driving and a lot of things I used to love to enjoy. (S64, MD)*

In response to not being able to drive many participants reported they accessed alternatives to driving such as getting a scooter and using public transport such as buses and trains or,”*Community Transport.” (S102, MD).*

*I have lost the sight of my right eye during an operation. I don't drive a car now; I have a scooter. (S90, C)*

*I have had macular degeneration for last 3 1/2 years. I now need magnifier to read. Have had to re-learn how to crossroads, catch buses, etc. (S95, MD)*

For some the decision to cease driving was made over a period as they experienced deteriorating vision or ailing health.

*I have Macular Degeneration which is increasing. Although I still have a driver's licence I rarely drive (can't see well enough). I mainly travel by public transport and in friends’ cars. (S64, MD)*

*I have macular degeneration (dry mildly). I don't walk as far as I used to, and I miss that. I had a serious fall late December and my shoulder is improving slowly. I've curtailed my driving (car). Actually, I am thinking of handing in my licence, and purchasing a 'golfer'. (S122, MD)*

A few participants reported that the experience of participation in a car accident led them to giving up their license, even when most reported they weren’t at fault.

*I have been severely depressed as my car was a complete write off. I haven't bought another car, as I haven't the confidence to drive anymore! (S180, C)*

Others struggled finding alternatives to driving and having to rely on others.

*We are frustrated by the lack of public transport, as I no longer wish to drive in busy cities. (S147, C)*

*The macular degeneration has stopped me driving my car and have to rely on others for transport as we don't have public transport. I spend most days at home. (S14, MD)*

*My biggest problem is my poor eyesight, because of which I have had to give up driving and now have to rely on my husband and family members and friends. Also because of my sight I need help shopping, etc. (S106, C)*

Lost independence was grieved by many and to the expereince of negative emotions such as altered mood and feelings of grief.

*My eye problems have become worse meaning I can’t drive any more. I have become more dependent because of this – I don't like this situation. (S38 MD)*

*I've had a really horrible year—so far. My independence has been taken away from me, because of a bad case for Macular Degeneration, blind within 2 days. Had to sell my car. (S5, MD)*

Participant also linked not being able to drive with significant social changes, especially feelings of greater social isolation due to not being able to visit people or engage within the community.

*Since last communication, I have lost my Driving Licence (after 60 years) I miss the easy availability and independence enormously and am now lonely and miserable and dependent on others so much. (S24, MD)*

*I have given up my car and this has taken away a lot of my independence. I now need to rely on others to get around and find this is restricting my social network. (S9, MD)*

However, the impact of driving cessation and feelings of isolation and lack of access to public transport were exemplified among those living in rural and remote setting.

*As I can no longer drive, I am very reliant, on others as I live 30 km from the town. Trips are few—far between. (S118, G)*

## Discussion

This study aimed to understand and characterise the experience of driving and driving cessation in older Australian women with self-reported eye disease. A central finding of this study was that successful treatment for eye disease allowed older women to continue driving. Driving was a highly valued activity that promoted community engagement and participants worried about how they would manage in the even they couldn’t drive in the future. With increased age participants with eye disease ceased driving due to deteriorating in vision or other health conditions. Treatment that ameliorates vision loss and blindness in a challenged for public health professionals given the association between the progressive decline of quality of life and vision loss [38] which is compounded by driving cessation.

Age-related eye disease, including glaucoma, macular degeneration, and diabetic retinopathy have a significant impact on the ageing population [39]. Early detection and treatment, such as access pharmacological treatments [40] and surgical treatments [41] can prevent lasting damage and improve driving performance [42]. The extent of benefit from treatment for eye disease is apparent in our study whereby, even in this oldest cohort of the ALSWH, many were able to continue to drive for a varying number of years.

Consistent with previous research older drivers in our study cited poor vision as a major factor in implementing driving restrictions and avoidance of potentially difficult driving situations [43]. Indeed, a central finding of this study was the that older adults were aware of and compensated for their declining vision by self-implementing driving restrictions in an effort to continue driving. This is consistent with findings from other driving studies [9, 44]. Driving restrictions helped maintain ADL performance such as the ability care for the others and provide a service to the community, such a volunteering as well maintaining social engagement. However, we also identified that reduced driving confidence was linked with driving avoidance, self-initiation of driving restrictions and driving cessation.

Poor vision is a known risk factor for driving cessation or avoidance and a dependence on others for transport, typically children and friends [45–47]. Participants in this study identified that decision to stop driving was often anticipated with apprehension and was not easy decision. Echoing with previous research driving cessation can led to negative emotions due to social isolation and relying on other [11]. However, a pleasing finding in this study was that some women readily transitioned to using public transport when they ceased driving. This is in contrast to previous literature suggesting that and older drivers do not make preparations ahead of driving cessation and do not plan for what they might need to do to meet their mobility needs [48, 49]. Prior studies suggest the need for drivers to plan ahead when making the decision not drive and that health professionals, such as occupational therapists, may play an important role in helping older adults prepare for this transition [49, 50]. The decision not to drive may also represent changes in overall functioning and we have previously identified that among the oldest old (persons aged 85 years or older), the difference between driving and not driving typically also reflects a sharp contrast in level of physical fitness and mental functioning [51].

Disease trajectories can provide insight into disease onset, progression, and resolution and therefore can assist with the planning and appropriately-timed delivery of relevant services [52]. Whillans et al. (2016) [53] undertook a longitudinal analysis over an eight year time frame regarding self-reported vision among 2956 respondents, aged 60 years and over, as part of the English longitudinal study of ageing. Finding demonstrated that the onset of diagnosed eye diseases was associated with trajectories of rapid deterioration in vision, fair vision, and poor vision. Participants with age-related macular degeneration were more likely to report gradual deterioration from good to fair and less likely to report stable good vision and respondents with diagnosed cataracts were more likely to report rapid deterioration from excellent to fair vision. Cataract surgery was positively associated with reporting stable excellent vision [53].. Patterns of driving following treatment for eye disease in our study echo with these findings.

The strength of this study lies in a mixed methods exploration of the experience of driving in older women. Participant’s free-text comments demonstrated a diversity of the expereince of driving over time due to eye disease and we achieved thematic saturation. We acknowledge study bias due to self-report, also many women who did not provide a comment and they may have differing experiences.

## Conclusion

Older Australian women have care needs specific with regards to their ability to drive pending their stage in life and diagnosis of eye disease. Key findings highlighted that deterioration in vision led to driving cessation or avoidance and a dependence on others for transport, typically children and friends. Overall access to successful treatment for eye disease allowed older women to continue driving however we also identified that driving confidence reduced over time.

## Data Availability

The datasets generated and/or analysed during the current study are not publicly available due to ethical and privacy constraints but are available from the corresponding author on reasonable request.

## Abbreviations

ADL: Activities of Daily Living
ALSWH: Australian Longitudinal Study on Women’s Health
C: Cataract
G: Glaucoma
MD: Macular Degeneration
